# 

**Published:** 1843-10-14

**Authors:** 


// THE MEDICAL EXAMINER,

### And Retrospect of the Medical Sciences.

EDITED BY

MERÉDITH CLYMER, M.D.

Lecturer on the Institutes of Medicine, Physician to the Philadelphia Hospital, Blockley, Fellow of the College of Physicians, Philadelphia, etc. etc.

VOL. VI.] PHILADELPHIA, OCTOBER 14, 1843. [No. 20.

